# An exploration of parents’ preferences for foot care in juvenile idiopathic arthritis: a possible role for the discrete choice experiment

**DOI:** 10.1186/1757-1146-7-10

**Published:** 2014-02-06

**Authors:** Gordon J Hendry, Debbie E Turner, Janet Gardner-Medwin, Paula K Lorgelly, James Woodburn

**Affiliations:** 1 School of Health & Life Sciences, Institute for Applied Health Research, Glasgow Caledonian University, Glasgow G4 0BA, UK; 2 Department of Child Health, University of Glasgow, Glasgow, UK; 3 Centre for Health Economics, Monash University, Clayton, Australia

**Keywords:** Juvenile idiopathic arthritis, Foot, Discrete choice experiment, Health economics, Podiatry

## Abstract

**Background:**

An increased awareness of patients’ and parents’ care preferences regarding foot care is desirable from a clinical perspective as such information may be utilised to optimise care delivery. The aim of this study was to examine parents’ preferences for, and valuations of foot care and foot-related outcomes in juvenile idiopathic arthritis (JIA).

**Methods:**

A discrete choice experiment (DCE) incorporating willingness-to-pay (WTP) questions was conducted by surveying 42 parents of children with JIA who were enrolled in a randomised-controlled trial of multidisciplinary foot care at a single UK paediatric rheumatology outpatients department. Attributes explored were: levels of pain; mobility; ability to perform activities of daily living (ADL); waiting time; referral route; and footwear. The DCE was administered at trial baseline. DCE data were analysed using a multinomial-logit-regression model to estimate preferences and relative importance of attributes of foot care. A stated-preference WTP question was presented to estimate parents’ monetary valuation of health and service improvements.

**Results:**

Every attribute in the DCE was statistically significant (*p* < 0.01) except that of cost (*p* = 0.118), suggesting that all attributes, except cost, have an impact on parents’ preferences for foot care for their child. The magnitudes of the coefficients indicate that the strength of preference for each attribute was (in descending order): improved ability to perform ADL, reductions in foot pain, improved mobility, improved ability to wear desired footwear, multidisciplinary foot care route, and reduced waiting time. Parents’ estimated mean annual WTP for a multidisciplinary foot care service was £1,119.05.

**Conclusions:**

In terms of foot care service provision for children with JIA, parents appear to prefer improvements in health outcomes over non-health outcomes and service process attributes. Cost was relatively less important than other attributes suggesting that it does not appear to impact on parents’ preferences.

## Background

Foot impairments and disability persist in over 60% of children who have juvenile idiopathic arthritis (JIA) despite recent improvements in its medical management [1,2]. The management of foot problems in JIA is complex and involves combinations of medical and non-medical therapies such as intra-articular corticosteroid injections, foot orthoses and exercise regimens [2-5]. Regular visits to out-patients clinics are often necessary and the burden of seeking care may be detrimental to routine family life [6]. Moreover, paediatric patient adherence to rehabilitative strategies is often poor and non-compliance may limit the potential for benefits in outcomes following therapy [7,8]. As such children with JIA may respond poorly to intervention resulting in sustained physical impairment, which may contribute negatively to the economic impact of JIA [9,10].

Health care priorities and the preferences of health care providers’ have been found to differ from those of their patients, particularly patients with inflammatory arthritis [11]. An increased awareness of patients’ and parents’ care preferences is desirable from a clinical perspective as such information may be utilised to optimise care delivery [12,13]. Qualitative perceptions of foot care in JIA have been explored recently and several areas for service improvement have been identified [14]. However a limitation of qualitative research is that it does not permit the ranking of preferences to provide information regarding the importance of different aspects of care.

Parents’ preferences for drug treatments and health outcomes in JIA have recently been explored, using a technique known as a discrete choice experiment (DCE) [15]. This study found that parents demonstrated stronger preferences for treatments that reduced pain and improved daily functioning, regardless of other considerations such as associated side effects [15]. A DCE is a quantitative technique for eliciting individual’s preferences for care [16]. A DCE questionnaire is comprised of choice sets of hypothetical scenarios that are presented to study participants. Each scenario describes different levels of the attributes that characterise the intervention under evaluation [17]. The participants’ preferences are elicited by asking them to state which scenario they prefer. DCEs are advantageous by several means; they are useful in the context of clinical trials, as they permit the gathering of rich preference information concerning interventions that are exploratory/experimental in nature [18,19]; they result in trade-off decisions between health-related attributes which mimic real-life decision-making situations [16,20]; and they can be used within the framework of cost benefit analyses (CBA) to estimate the value of individuals’ care preferences [21,22]. When cost is also considered as an attribute alongside health-related outcomes, it is possible to conduct an indirect calculation of respondents’ willingness-to-pay (WTP) for health care [23,24]. Willingness-to-pay can be formally defined as the maximum amount of money that an individual is prepared to part with in order to receive a particular service [25]. The WTP concept has been applied previously in JIA to assess health care preferences of families of children with the disease using a series of ‘bidding game’ questions [26], but it may also be elicited through face to face interviews or self-administered questionnaires.

To understand parents’ preferences, two elicitation techniques were employed. A DCE provided insight as to the relative importance of various attributes of foot care. Additionally willingness-to-pay (WTP) values were elicited to understand the monetary value parents place on foot care for children/adolescents with JIA.

## Methods

### Participants and setting

The DCE here was embedded within an exploratory, non-pharmacological randomised controlled trial (RCT) designed to investigate the effectiveness of a new multidisciplinary foot care programme for children/adolescents with JIA and disease-related foot problems [27,28]. Study participants included parents/guardians of children with JIA who met the inclusion criteria for the RCT [27,28] which took place at the Royal Hospital for Sick Children, Glasgow, UK between March 2009 and March 2011. Briefly, children/adolescents with JIA were included if they had a documented history of active foot disease. The DCE was administered at baseline of the RCT. This RCT was registered with the International Standard Randomised Controlled Trial Number (ISRCTN) register (registration number ISRCTN49672274). The Glasgow West Local Research Ethics Committee granted ethical approval for this study (Ref 06/S0703129). Written informed consent was obtained from participants in accordance with the Declaration of Helsinki.

### DCE development

DCEs are comprised of choice sets of hypothetical scenarios. Each scenario includes a set of attributes, at varying levels, that characterise the intervention or healthcare service under evaluation [17]. For example; the attribute “Waiting time for appointment” could be described by the levels “1 month”, “3 months” or “6 months”. These levels were agreed upon through consensus within the trial steering committee based upon the local centre’s waiting times for podiatry contact (3-6 months) which we determined through a pilot study [2]. The DCE presents a number of scenarios, where the attribute levels vary, and participants are asked to state which scenario they prefer. To identify the attributes that might be important, a literature review was first conducted [29]. This then informed the design of a qualitative study of foot problems and foot care in JIA, whereby the DCE attributes were derived from the thematic analysis of the focus group and interview data [14]. The attributes identified as important to JIA sufferers were: pain, mobility impairment, reduced ability to perform activities of daily living (ADL), footwear difficulties, and poor referral pathways/delayed access to care (waiting) (see Table 1) [14]. The route to podiatry care, particularly the role of a multidisciplinary team, was also included as an attribute as this was the intervention in the trial. Cost was included to allow for an estimation of the marginal WTP of each attribute. The lowest cost level was set at £80 by consensus within the trial steering committee and was based upon inflated costs of a single UK National Health Service podiatry consultation [30], with £70 increments selected for the remaining two cost levels (£150 and £220). Note that a maximum of eight attributes were targeted a priori to reduce cognitive burden [16,31,32].

**Table 1:** Description of final attributes and levels in the hypothetical scenarios

**Attribute**	**Description**	**Variable names and levels**
Pain relief	Following treatment you will have the following pain level	pain_0; no lower limb pain experienced whatsoever, pain_1; A noticeable improvement in lower limb pain, pain_2; no change in the levels of pain
Improvement in mobility	Following treatment you will have the following ability	mobility_0; Ability to move freely, mobility_1; a noticeable improvement in the ability to move but some limitations, mobility_2; No change in movement ability
Activities of daily living	Following treatment you will have the following ability	adl_0; Ability to take part in all usual everyday activities, adl_1; Ability to take part in some usual everyday activities, adl_2; No improvement in ability
Route to podiatry care (foot care)	You will receive podiatry care via	route_0; The appointment includes seeing the consultant, the podiatrist and the physiotherapist in the same visit, route_1; The consultant would decide whether to refer you/your child to the podiatrist
Waiting time	The waiting time for first podiatry contact will be	Wait; 3months, 6months, 1 year.
Footwear	Following treatment you will be able to wear	footwear_0; ability to wear *most types of shoes*, footwear_1; ability to wear *limited types of shoes*, footwear_2; ability to wear *specially made* shoes only
Cost to you	The total cost of the appointment to you will be	Cost; £80, £150, £220.

**Figure 1:**
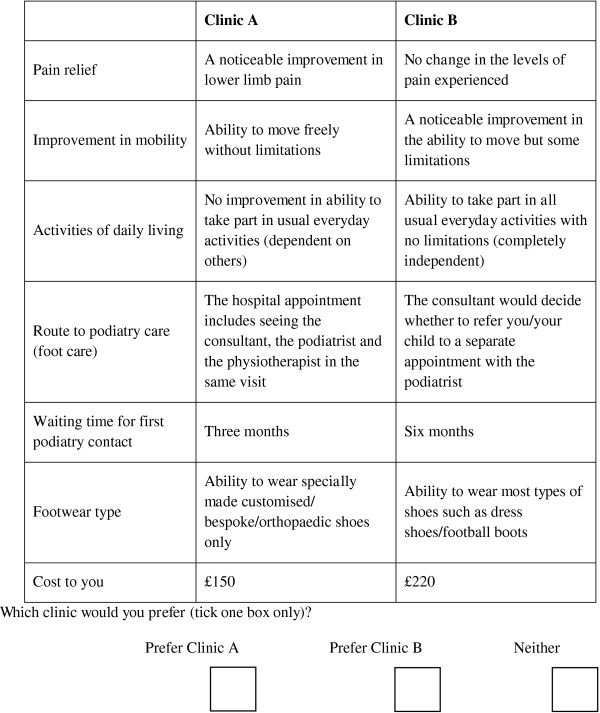
**An extract choice set from the final DCE questionnaire.** Eighteen of these choice sets were presented in the final questionnaire.

The combination of attributes and levels, resulted in large number of unique scenarios (six attributes with three levels, one attribute with two levels = 1458 possible scenarios), such that it was necessary to estimate a smaller fractional factorial design. The final attributes and levels were formulated into 18 hypothetical scenarios and were tested against optimum efficiency design criteria using the SPExpt software package [33]. This software calculates various potential fractional factorial designs based upon the full factorial design, permitting researchers to choose a feasible number of choice scenarios for a DCE questionnaire. The fractional design was found to be 100% efficient, meeting the following criteria; orthogonal with level balance (each attribute level in the design occurs equally often, thus mimicking a full factorial design), minimal overlap (attribute levels are sufficiently varied in order to allow meaningful comparison and trade-off, thus increasing the questionnaire’s ability for eliciting the maximum amount of information possible from each respondent), and uncorrelated main effects (the attributes of the design as statistically independent of one another and therefore the strength of preference elicited from participants are not violated by aspects of the design) [16,19]. Scenarios were paired using a fold-over technique to create 18 pair-wise choices, where each scenario was presented to respondents as “mirrored images” of the alternative scenario (therefore maintaining 100% design efficiency) [34]. Each choice set consisted of two alternative hypothetical foot care programme scenarios (A or B). A ‘neither’ option was also included to offer to more closely resemble a real world context (that is parents can choose not to have their child treated) (see Figure 1) [16,35].

### WTP elicitation

A payment scale (stated preference) WTP question, with the addition of an open-ended question for large values, was included to the start of the questionnaire. Respondents were asked to indicate how much they would pay (0-£400, in £20 and then £50 increments, or some higher value) for an ‘ideal’ clinical scenario derived by the RCT steering committee, which was effectively all the highest levels of the DCE attributes (except cost).

### Statistical analysis

DCE data were entered analysed using Stata Release 11 (StataCorp LP, Texas, USA) using a fixed effects multinomial logit regression model based on McFadden’s random utility model [36]. The ordinal variables ‘cost’ and ‘waiting time’ were re-coded as continuous variables. Dummy coding was used to describe all remaining variables [37]. Thus all ‘no change’ level selections for attributes; pain, mobility, activities of daily living (ADL) and footwear were excluded from the analysis as they represented the status quo. Therefore the regression analysis was conducted to calculate beta (β) coefficients for ‘maximum improvement’ (for example; pain_0, ADL_0) or a ‘noticeable improvement’ (for example; pain_1, ADL_1) attribute levels. The sign of the β coefficient (positive or negative) indicates the direction of preference. For example, a positive β coefficient occurs where an increase in the attribute level results in an increased likelihood that the study participant will choose that scenario based on their preference for an increase in the level of that attribute [38]. The magnitude of the β coefficient represents the strength of preference for choosing that particular attribute. The marginal WTP for improvements in individual attributes were to be calculated by dividing the coefficient of interest by the coefficient attached to ‘cost’, pending the statistical significance of this attribute following regression analysis [21].

Stated preference WTP values for the hypothetical multidisciplinary foot clinic derived from the payment scale WTP question are reported as mean values. The average annual WTP for the hypothetical ‘ideal’ scenario was calculated by multiplying the mean WTP estimate by the mean number of clinical consultations per participant over the 12 months of the RCT. As such, the WTP for a single clinical visit was multiplied by 5 (there were 5 clinical visits over the 12months of the RCT) to provide an estimate of annual WTP. This health care resource use/consumption data was collected retrospectively from patients’ case notes after final follow up in the main RCT (12 months from baseline).

## Results

### Respondents

Forty-two parents (38 female: 4 male) of 42 children (29 female: 13 male) with a mean (SD) age of 10.1 (3.81) returned completed questionnaires from the 44 issued at trial baseline, giving a response rate of 95% (see Table 2 for parent RCT participant baseline characteristics). Two female parent participants did not complete the questionnaire (one parent refused to participate stating the distressing nature of choosing between health states for their child as the primary reason, one parent did not return their completed questionnaire). As each participant (n = 42) was provided with 18 hypothetical choice set scenarios in the DCE, there is a total of 756 observations. For 141 (18.7%) of these, respondents selected the ‘neither’ option. Therefore, there were 615 (81.3%) of 756 observations where respondents indicated a preference for scenarios A or B.

**Table 2:** **Baseline characteristics of the parent RCT**[23]**participants (n = 42 children/adolescents)**

**Characteristic**	**Value**
**Demographics**	
Age (yrs)*	10.1 (3.8)
Male/female (n)	13/29
Body mass index*	19.1
SDS BMI percentiles*	64.5 (0.3)
Disease duration (months)*	46.9 (35.9)
**Pharmacological management**	
Analgesics: n (%)	5 (12)
NSAIDs: n (%)	5 (12)
Methotrexate: n (%)	32 (76)
Etanercept: n (%)	12 (29)
Sulphasalazine: n (%)	1 (2)
Rituximab: n (%)	1 (2)
Combination methotrexate & etanercept: n (%)	10 (24)
**Disease subtypes**	
Persistent oligo: n (%)	10 (24)
Extended oligo: n (%)	9 (21)
Poly-: n (%)	15 (36)
Poly+: n (%)	2 (5)
PsA: n (%)	3 (7)
ERA: n (%)	2 (5)
Systemic: n (%)	0 (0)
Undifferentiated: n (%)	1 (2)

*Mean (standard deviation); n, number of participants; SDS, standardised deviation score British 1990 growth reference (UK 90); NSAID, non-steroidal anti-inflammatory drugs; Poly -, polyarthritis rheumatoid factor negative; poly+, polyarthritis rheumatoid factor positive; PsA, psoriatic arthritis; ERA, enthesitis related arthritis.

### Relative importance of attributes

The frequency of observations for the majority of each attribute levels suggests that the DCE and regression model appeared to be internally valid; respondents prefer at least noticeable improvements in health based outcomes (see Table 3). Although the observations for ‘wait’ appeared to be contrary to *a priori* expectations, as some respondents appeared to choose a longer waiting time. In 37.2% of observations respondents chose a scenario with a 3 month waiting time, while in 32% of observations respondents chose a scenario with a 12 months waiting time versus 30.7% of observations where respondents chose a scenario with a 6 months waiting time. Similarly, observations for ‘cost’ appeared to be contrary to *a priori* expectations as respondents tended to choose a scenario with a greater cost (in 30.08% of observations respondents chose £80, in 31.71% of observations respondents chose a scenario with £150, and in 38.21% of observations respondents chose a scenario with £220.

**Table 3:** Frequencies (absolute and cumulative %) of observations for each attribute level

**Attribute**	Levels*	**Frequency (abs)**	**Frequency (%)**	**Cum frequency (%)**
**Pain**	pain_0	250	40.65	40.65
	pain_1	219	35.61	76.26
	pain_2	146	23.74	100.00
**Mobility**	mobility_0	246	40.00	40.00
	mobility_1	222	36.10	76.10
	mobility_2	147	23.90	100.00
**ADL**	adl_0	292	47.48	47.48
	adl_1	187	30.41	77.89
	adl_2	136	22.11	100.00
**Route**	route_0	345	56.10	56.10
	route_1	270	43.90	100.00
**Wait**	3 months	229	37.24	37.24
	6 months	189	30.73	67.97
	12 months	197	32.03	100.00
**Footwear**	footwear_0	250	40.65	40.65
	footwear_1	206	33.50	74.15
	footwear_2	159	25.85	100.00
**Cost**	£80	185	30.08	30.08
	£150	195	31.71	61.79
	£220	235	38.21	100.00

*pain_0; no lower limb pain; pain_1 a noticeable improvement, pain_2; no change, mobility_0; ability to move freely, mobility_1; a noticeable improvement; mobility_2; no change; adl_0 ability to take part in all activities, adl_1; ability to take part in some activities, adl_2 no improvement, route_0; appointment including the consultant, podiatrist and physio, route_1; consultant’s decision to refer, waiting time; 3months, 6months, 12months, footwear_0; ability to wear most shoes, footwear_1; ability to wear limited shoes, footwear_2; custom shoes only, cost; £80, £150, £220.

### Preferences for attributes of care

**Figure 2:**
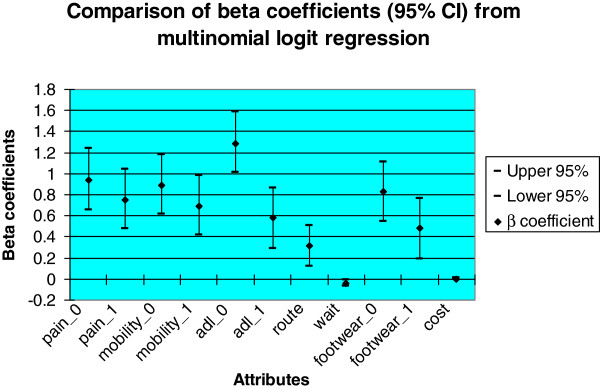
Error bars showing beta coefficients and associated 95% confidence intervals derived from fixed effects multinomial logit regression analysis.

Each attribute’s regression coefficients (β) were statistically significant (*p* < 0.01) except cost (β = 0.002, *p* = 0.118), suggesting that all attributes, except cost, were independently associated with parent respondents’ preferences (see Table 4). For the entire cohort of respondents, the magnitudes of the coefficients indicate that the order of importance (that is strength of preference) for each attribute level was: ‘ability to take part in all activities’ (β = 1.29), ‘no lower limb pain’ (β = 0.94), ‘ability to move freely’ (β = 0.89,), ‘ability to wear most shoes’ (β = 0.83), ‘a noticeable improvement in pain’ (β = 0.75), ‘a noticeable improvement in mobility’ (β = 0.69), ‘ability to take part in some activities’ (β = 0.58), ‘ability to wear limited shoes’ (β = 0.48), ‘appointment including consultant, podiatrist and physio’ (β = 0.31), and ‘waiting time’ (β = -0.04) (see Table 4 and Figure 2). The sign of the β values suggests that parents preferred: a reduction in pain, improvements in mobility, the ability to perform ADL, and the ability to wear desired footwear; referral to a multi-disciplinary foot-care programme; and a reduced waiting time.

**Table 4:** Results from the fixed effect multinomial logit regression

Attributes*	**β coefficient**	**SE**	**95% CI**	**p-value**
pain_0	0.94	0.15	(0.65, 1.24)	p < 0.01
pain_1	0.75	0.15	(0.47, 1.04	p < 0.01
mobility_0	0.89	0.15	(0.61, 1.18)	p < 0.01
mobility_1	0.69	0.14	(0.41, 0.98)	p < 0.01
adl_0	1.29	0.14	(1.01, 1.58)	p < 0.01
adl_1	0.58	0.15	(0.29, 0.86)	p < 0.01
Route	0.31	0.09	(0.12, 0.5)	p < 0.01
Wait**	−0.04	0.02	(-0.07, -0.008)	p = 0.013
footwear_0	0.83	0.14	(0.54, 1.11)	p < 0.01
footwear_1	0.48	0.14	(0.19, 0.76)	p < 0.01
Cost**	0.002	0.001	(-0.0005, 0.004)	p = 0.138 NS

*pain_0; no lower limb pain; pain_1 a noticeable improvement, mobility_0; ability to move freely, mobility_1; a noticeable improvement; adl_0 ability to take part in all activities, adl_1; ability to take part in some activities, route; appointment including the consultant, podiatrist and physio, footwear_0; ability to wear most shoes, footwear_1; ability to wear limited shoes.

**continuous variables.

### Willingness to pay

The statistical non-significance of the cost attribute meant that it was inappropriate to estimate indirect WTP values using the DCE. From the stated preference WTP question the mean (SD) WTP for the hypothetical clinical scenario was £223.81 (£144.37), and the median (range) was £200.00 (£0-£400). Nine respondents (21%) indicated that they would pay up to £80, 15 respondents (36%) indicated that they would pay between £100 and up to and including £200, 18 respondents (43%) indicated that they would pay £220 or more. Of the latter 18 respondents, 7 stated that they would pay more than £400 for the hypothetical clinical scenario. There were five clinical trial appointments per participant over the 12 months of the RCT, therefore parents’ mean annual WTP for the intervention was estimated as £1,119.05. Two participants stated that they ‘could not afford to pay’ for the hypothetical clinic, but if they ‘could pay they probably would’. Two participants stated that they ‘would object to paying any extra on top of tax’ for the hypothetical clinic. Six participants in total stated that they could not put a limit on what they would pay because they would ‘pay anything’. One participant stated that they would pay £500 for the ‘ideal’ clinical scenario.

**Figure 3:**
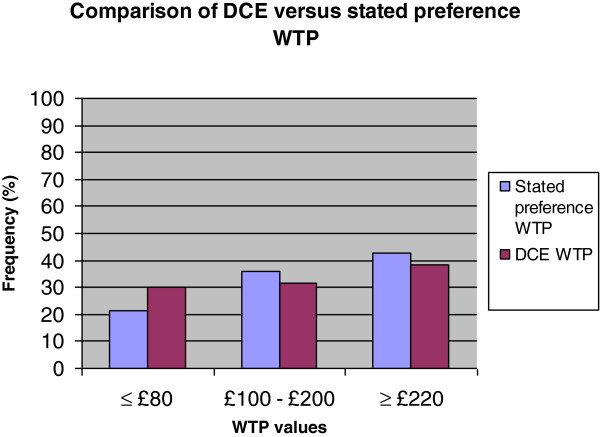
Comparison of the % frequency of n = 42 stated preference WTP values versus n = 615 DCE WTP observations from the same respondents.

From the DCE observations (n = 615), 30% of responses indicated a preference for a cost of £80, 32% for a cost of £150, and 38% for £220. Aggregated scores from the stated preference WTP question responses (n = 42) indicated that 21% of respondents were prepared to incur a cost of up to £80, 36% for between £100-£200, and 43% for £220 or more for the hypothetical clinical scenario (Figure 3). The stated preference WTP responses are relatively consistent with the DCE observations suggests that the DCE model was internally valid, as respondents appeared to be WTP a higher cost for improvements in foot health and process attributes regardless of elicitation method.

Parents’ WTP for 12 months of the experimental intervention was estimated at £1,119.05, derived from the stated preference WTP question and hypothetical ‘ideal’ scenario. This was far lower than the estimated mean annual cost of the experimental intervention that was subject to investigation via the RCT which was £5,025.70. Estimated costs of the intervention exceeded parents’ perceived valuation of benefits of the best possible outcome.

## Discussion

This study resulted reports on the development and design of a DCE and stated preference WTP questionnaire used to elicit parental preferences for foot care for children with JIA. According to the British Society for Paediatric and Adolescent Rheumatology (BSPAR) and Arthritis and Musculoskeletal Alliance (ARMA) standards of care for JIA guidelines, all children and adolescents with JIA should have access to foot care [39,40]. At present very little is known about the provision of foot care in JIA [2,14], and there is little evidence on parents’ views and opinions of foot care, particularly from those who have had no experience of foot care services. As such this study adds rich and robust information regarding preferences for foot care in JIA which could be utilised to improve foot care services.

The results suggest that parent respondents appeared to prefer improvements in health outcomes over non-health outcomes and podiatry service process attributes. This is a common but not an exclusive finding in DCE research. A recent DCE found that parents of children with JIA demonstrated strong preferences for interventions that reduced pain and improved function [15]. In contrast, adult rheumatology outpatients ranked non-health attributes as more important relative to pain levels [41]. The results from this study may represent parents’ desires for their children to have more normal lives, but further research is required to substantiate this claim.

Maximal improvements in the ADL attribute appeared to be the most important attribute to parents. Arguably this finding is somewhat surprising, as it appears to have a greater influence over parents’ choices relative to improvements in pain relief. Pain relief has been cited an important aspect of care by parents, patients, paediatric rheumatologists and allied health professionals in previous studies [14,15]. Overall, maximal improvements in the ADL attribute, together with ‘pain’, ‘mobility’ and ‘ability to wear desired footwear’ attributes were considered to be most important to the parent respondents. However, it is possible that children’s experiences of JIA may lead to different preferences for care that are centred on their sense of self and social identity [42]. For example, in this study parents appeared to prefer the options for improvements in their child’s health, however qualitative research suggests children with JIA may not want to be labelled as sick or disabled and therefore may have entirely different preferences for their own care [42].

The parental preference data derived from this DCE may be useful for informing aspects of service provision for children who have JIA related foot problems. Previous research suggests paediatric rheumatologists and health professionals require more information about which foot symptoms are in need of urgent attention [14]. Parental preferences for the key outcomes identified in this study could be incorporated into a new patient-reported outcome measure designed to inform urgency for referral to foot care services. Alternatively, the use of an existing tool known as the Juvenile Arthritis Foot Disability Index [43] which includes items related to these key outcomes, could be re-designed to incorporate foot disability severity thresholds and guide referrals for foot care.

A lack of suitable footwear choices for children with JIA-related foot disease has been previously identified as problematic [14]. Parents appeared to rank the ability of their child to wear desired footwear as important, confirming previous findings. In contrast to a growing body of research in adult RA, [44,45] the impact of limited footwear choices as a result of disease-related foot problems have not been investigated in JIA. This is surprising as foot deformities such as valgus hindfoot and clawed lesser toes appear to be common [2,46,47].

Of the process attributes, ‘route to care’ involving an appointment with the paediatric rheumatologist, podiatrist and physiotherapist in the same visit was considered to some importance, and was more important than waiting time and cost. This is a novel finding as it demonstrates that parents may gain utility from the prospect of their child receiving multi-disciplinary foot care, as opposed to a separate doctor’s referral for separate foot care. However, this could be the result of the Hawthorne effect where respondents have modified their decision making process as a response to being enrolled in the main ‘open label’ RCT of multidisciplinary foot care [23,48].

Unlike the majority of DCEs, the cost attribute did not reach statistical significance in the regression analysis. This implies that parents’ preferences were not influenced by having to pay more. The stated preference (payment scale) WTP question supports this, on average parents were willing to pay £224, with six parents stating that they would pay anything to improve their child’s health outcomes. It is important to acknowledge that in a health care system where care is free at the point of access (as is the case in the NHS); it is difficult to obtain accurate information to inform the levels of cost in a DCE. It is possible that the values of £80, £150 and £220 were too low. In addition, household income data was not available and as such WTP figures were not expressed as a percentage of income, which would have been a more useful representation of parents’ WTP. Future research perhaps should seek to replicate this DCE with higher values as guided by the stated preference WTP results, or preferably as a percentage of household income. Only then would it be possible to ascertain if a parent views their child’s health as ‘priceless’.

When completing DCEs, respondents are required to process large amounts of information resulting in significant cognitive burden [49]. As such, respondents may make attempts to reduce their cognitive burden through lexicographic and heuristic strategies [26,50,51]. Lexicographic preferences are where a respondent ranks attributes in order of importance and bases their decision on the highest priority attributes [49]. While heuristics are where respondents employ simple ‘rules of thumb’ to reduce the cognitive burden, such as limiting their responses to trade-offs between one or two attributes per scenario [50,51]. Furthermore, the sequential ordering of attributes in the DCE scenarios can have an effect on the relative utility weights associated with those attributes [52]. The effects of sequential ordering on utility weights were not formally explored in this study; however it is interesting to note that the β coefficients of the first three attributes listed in the DCE scenarios have the greatest magnitude. Whereas, ‘cost’ was the lowermost attribute in the DCE scenarios and had the smallest β coefficient magnitude.

The sample size of the study must also be considered when interpreting its results. The sample size was dictated by the number of participants enrolled in the main RCT [27]. It should also be noted this DCE was performed in a group of parents whose children were enrolled in an exploratory RCT of multidisciplinary foot care [27] and therefore there may be a threat to the external validity of its findings due to cross-purpose, convenience and Hawthorne effects. Nevertheless, the DCE was administered at baseline prior to exposure to the foot care intervention, and the inclusion criteria of the parent study [27] (see Participants and Setting) appear directly relevant to the present study which aimed to elicit foot care preference information of parents of children with JIA who have foot problems. Of those parents who participated, only 42 fully completed the DCE questionnaire. According to the literature previous DCEs have recruited a minimum of 40 respondents to successfully establish sufficient models [53]. However with the inclusion of the ‘neither’ option and the high frequency of non-responses, it is likely that a larger sample size may have improved both the richness and robustness of the results. It should be noted that the sample size was driven by the RCT sample which, while not directly relevant to the statistical modelling in this study was appropriately powered for the trial endpoint.

An unresolved issue in health economics is the application of WTP elicitation methods where respondents receive care which is free at the point of contact in a publicly funded healthcare system such as the NHS in the UK. As such some respondents may interpret the WTP value as the cost incurred by the health service to deliver that service. Alternatively respondents may interpret the cost attribute as one that can be ignored as cost is not incurred by them directly [21]. It is possible that either of these points may have contributed to the non-significance of the cost attribute in this study. Lastly, it should be noted that household income data was not measured as part of this study so as not to overburden respondents who were required to complete many questionnaires during the main RCT. This restricted our ability to identify whether or not income influenced respondents choice, particularly concerning cost. As such, the results relating to cost could be potentially misleading, and future DCEs including a cost attribute should include cost as an appropriately determined percentage of household income.

## Conclusions

This study is the first study to employ a DCE to elicit preferences from parents of children with JIA regarding foot care. The health attributes ‘ADL’, ‘pain’, ‘mobility’, and ‘footwear’, as well as the non-health attributes ‘waiting time’ and ‘route to care’ should be considered by policy makers and health professionals involved in foot care services/care delivery. This study has uniquely highlighted the importance of non-health attributes of foot care to parents of children with JIA. Furthermore, it has demonstrated that parents appear to value, and gain utility from the idea of the potential benefits of foot care for their children. In a constrained economy with competing resources, this exploratory study suggests that decision makers might wish to favour interventions that improve ADL, pain and mobility, as these are valued most highly by parents.

## Competing interests

The authors declare that they have no competing interest.

## Authors’ contributions

GJH and PKL conceived and executed the study protocol. All co-authors contributed to the final design of the study protocol. GJH was responsible for the collection of study data. GJH and PKL interpreted the findings with assistance from all co-authors. GJH drafted the manuscript and the final version was read, reviewed and approved by all co-authors.

## References

[B1] Dekker M Hoeksma AF Dekker JHM van Rossum MAJ Dolman KM Beckerman H Roorda LD Strong relationships between disease activity, foot-related impairments, activity limitations and participation restrictions in children with juvenile idiopathic arthritis Clin Exp Rheumatol 2010 28 905 911 21122275

[B2] Hendry G Gardner-Medwin J Watt GF Woodburn J A survey of foot problems in juvenile idiopathic arthritis Musculoskel Care 2008 6 4 221 232 10.1002/msc.134 18618460

[B3] Cahill AM Cho SS Baskin KM Beukelman T Cron RQ Kaye RD Towbin RB Benefit of fluoroscopically guided intraarticular, long-acting corticosteroid injection for subtalar arthritis in juvenile idiopathic arthritis Pediatr Radiol 2007 37 544 548 10.1007/s00247-007-0457-6 17437095

[B4] Beukelman T Arabshahi CAM Kaye RD Cron RQ Benefit of intraarticular corticosteroid injection under fluoroscopic guidance for subtalar arthritis in juvenile idiopathic arthritis J Rheumatol 2006 33 11 2330 2336 16981290

[B5] Powell M Seid M Szer IS Efficacy of custom foot orthotics in improving pain and functional status in children with juvenile idiopathic arthritis: a randomized trial J Rheumatol 2005 32 943 950 15868634

[B6] Barlow JH Shaw KL Harrison K Consulting the ‘experts’: children’s and parents’ perceptions of psycho-educational interventions in the context of juvenile chronic arthritis Health Educ Res 1999 14 5 597 610 10.1093/her/14.5.597 10510068

[B7] April KT Feldmann DE Zunzunegui MV Duffy CM Association between perceived treatment adherence and health-related quality of life in children with juvenile idiopathic arthritis: perspectives of both parents and children Patient Prefer Adherence 2008 2 121 128 19920952 PMC2770382

[B8] Feldman DE De Civita M Dobkin PL Malleson PN Meshefedjian G Duffy CM Effects of adherence to treatment on short-term outcomes in children with juvenile idiopathic arthritis Arthritis Rheum 2007 57 6 905 912 10.1002/art.22907 17665485

[B9] Thornton J Lunt M Ashcroft DM Baildam E Foster H Davidson J Gardner-Medwin J Beresford MW Symmons D Thomson W Elliott RA Costing juvenile idiopathic arthritis: examining patient-based costs during the first year after diagnosis Rheumatology (Oxford) 2008 47 985 990 10.1093/rheumatology/ken039 18417528 PMC2430220

[B10] Bernatsky A Duffy C Malleson P Feldman DE St Pierre Y Clarke AE Economic impact of juvenile idiopathic arthritis Arthritis Rheum 2007 57 1 44 48 10.1002/art.22463 17266088

[B11] Kirwan JR Hewlett SE Heiberg T Hughes RA Carr M Hehir M Kvien TK Minnock P Newman SP Quest EM Taal E Wale J Incorporating the patient perspective into outcome assessment in rheumatoid arthritis--progress at OMERACT 7 J Rheumatol 2005 32 11 2250 2256 16265712

[B12] Heiberg T Kvien TK Preferences for improved health examined in 1,024 patients with rheumatoid arthritis: pain has highest priority Arthritis Care Res 2002 47 4 391 397 10.1002/art.10515 12209485

[B13] Holman H Lorig K Patients as partners in managing chronic disease BMJ 2000 320 7234 526 527 10.1136/bmj.320.7234.526 10688539 PMC1117581

[B14] Hendry GJ Turner DE Lorgelly PK Woodburn J Room for improvement: patients, parental and practitioners’ perceptions of foot problems and foot care in juvenile idiopathic arthritis Arch Phys Med Rehabil 2012 93 2062 2067 10.1016/j.apmr.2012.07.007 22842484

[B15] Burnett HF Regier DA Feldman BM Miller FA Ungar WJ Parents’ preferences for drug treatments in juvenile idiopathic arthritis: a discrete choice experiment Arthritis Care Res 2012 64 9 1382 1391 10.1002/acr.21698 22504893

[B16] Mangham LJ Hanson K McPake B How to do (or not to do) … Designing a discrete choice experiment for application in a low-income country Health Policy Plan 2009 24 2 151 158 10.1093/heapol/czn047 19112071

[B17] Skjoldborg US Lauridsen J Junker P Reliability of the discrete choice experiment at the input and output level in patients with rheumatoid arthritis Value Health 2009 12 1 153 158 10.1111/j.1524-4733.2008.00402.x 19911446

[B18] King MT Hall J Lancsar E Fiebig D Hossain I Louviere J Reddel HK Jenkins CR Patients preferences for managing asthma: results from a discrete choice experiment Health Econ 2007 16 703 717 10.1002/hec.1193 17238221

[B19] Viney R Lancsar E Louviere J Discrete choice experiments to measure consumer preferences for health and healthcare Expert Rev Pharmacoecon Outcomes Res 2002 2 4 89 96 10.1586/14737167.2.4.319 19807438

[B20] McIntosh E Donaldson C Ryan M Recent advances in the methods of cost-benefit analysis in healthcare: matching the art to the science Pharmacoeconomics 1999 15 357 367 10.2165/00019053-199915040-00003 10537954

[B21] Ratcliffe J The use of cojoint analysis to elicit willingness-to-pay values Int J Technol Assess Health Care 2000 16 1 270 275 10.1017/S0266462300161227 10815371

[B22] Ryan M Hughes J Using conjoint analysis to assess women’s preferences for miscarriage management Health Econ 1997 6 3 261 273 10.1002/(SICI)1099-1050(199705)6:3<261::AID-HEC262>3.0.CO;2-N 9226144

[B23] Ratcliffe J Van Haselen R Buxton M Hardy K Colehan J Partridge M Assessing patients’ preferences for characteristics associated with homeopathic and conventional treatment of asthma: a conjoint analysis study Thorax 2002 57 6 503 508 10.1136/thorax.57.6.503 12037224 PMC1746347

[B24] San Miguel F Ryan M McIntosh E Applying Conjoint Analysis in Economic Evaluations: an Application to Menorrhagia Appl Econ 2000 32 7 823 833 10.1080/000368400322165

[B25] Gafni A Willingness to pay in the context of an economic evaluation of healthcare programs: theory and practice Am J Manag Care 1997 3 S21 S32 10180338

[B26] Barron AC Lee TL Taylor J Moore T Passo MH Graham TB Griffin TA Grom AA Lovell DJ Brunner HI Feasibility and construct validity of the parent willingness-to-pay technique for children with juvenile idiopathic arthritis Arthritis Rheum 2004 51 6 899 908 10.1002/art.20829 15593249

[B27] Hendry GJ Watt GF Brandon M Friel L Turner DE Lorgelly PK Gardner-Medwin J Sturrock RD Woodburn J The effectiveness of a multidisciplinary foot care program for children and adolescents with juvenile idiopathic arthritis: and exploratory trial J Rehabil Med 2013 45 467 476 10.2340/16501977-1130 23571642

[B28] Hendry G Turner D McColl J Lorgelly P Sturrock R Watt G Browne M Gardner-Medwin J Friel L Woodburn J Protocol for the Foot in Juvenile Idiopathic Arthritis trial (FiJIA): a randomised controlled trial of an integrated foot care programme for foot problems in JIA J Foot Ankle Res 2009 2 1 21 10.1186/1757-1146-2-21 19566941 PMC2714841

[B29] Coast J Horrocks S Developing attributes and levels for discrete choice experiments using qualitative methods J Health Serv Res Policy 2007 12 1 25 30 10.1258/135581907779497602 17244394

[B30] UK Government Department of Health Available: https://www.gov.uk/government/publications/nhs-reference-costs-2012-to-2013 [accessed 10.01.14]

[B31] Lancsar E Louviere J Conducting discrete choice experiments to inform healthcare decision making: a users guide Pharmacoeconomics 2008 26 661 677 10.2165/00019053-200826080-00004 18620460

[B32] De Shazo JR Fermo G Designing choice sets for stated preference methods: the effects of complexity on choice consistency J Environ Econ Manage 2002 44 1 123 143 10.1006/jeem.2001.1199

[B33] Burgess L Discrete Choice Experiments 2013 Sydney: University of Technology [http://crsu.science.uts.edu.au/choice/choice.html]

[B34] Street DJ Burgess L Optimal stated preference choice experiments when all choice sets contain a specific option Stat Methodol 2004 1 1–2 37 45

[B35] Ryan M Skåtun D Modelling non-demanders in choice experiments Health Econ 2004 13 4 397 402 10.1002/hec.821 15067675

[B36] McFadden D Econometric models of probabilistic choice among products J Bus 1980 53 3 S13 S29

[B37] Bech M Gyrd-Hansen D Effects coding in discrete choice experiments Health Econ 2005 14 1079 1083 10.1002/hec.984 15852455

[B38] Gidman W Elliott R Payne K Meakin GH Moore J A comparison of parents and pediatric anesthesiologists? Preferences for attributes of child daycase surgery: a discrete choice experiment Paediatr Anaesth 2007 17 11 1043 1052 10.1111/j.1460-9592.2007.02271.x 17897269

[B39] Davies K Cleary G Foster H Hutchinson E Baildam E BSPAR standards of care for children and young people with juvenile idiopathic arthritis Rheumatology (Oxford) 2010 49 1406 1408 10.1093/rheumatology/kep460 20173199

[B40] Arthritis and Musculoskeletal Alliance (ARMA) Standards of Care for People with Inflammatory Arthritis 2010 http://arma.uk.net/wp-content/uploads/pdfs/musculoskeletalfoothealthproblems.pdf

[B41] Ryan M Bate A Eastmond CJ Ludbrook A Use of discrete choice experiments to elicit preferences Qual Health Care 2001 10 S1 i55 i60 11533440 10.1136/qhc.0100055.. PMC1765744

[B42] Tong A Jones J Craig JC Singh-Grewal D Children’s experiences of living with juvenile idiopathic arthritis: a thematic synthesis of qualitative studies Arthritis Care Res 2012 64 9 1392 1404 10.1002/acr.21695 22504867

[B43] Andre M Hagelberg S Stenstrom CH The juvenile arthritis foot disability index: development and evaluation of measurement properties J Rheumatol 2004 31 12 2488 2493 15570656

[B44] Williams AE Rome K Nester CJ A clinical trial of specialist footwear for patients with rheumatoid arthritis Rheumatology (Oxford) 2007 46 302 307 16877461 10.1093/rheumatology/kel234

[B45] Sylvester RN Williams AE Dalbeth N Rome K ‘Choosing shoes’: a preliminary study into the challenges facing clinicians in assessing footwear for rheumatoid patients J Foot Ankle Res 2010 3 24 10.1186/1757-1146-3-24 20959016 PMC2967518

[B46] Spraul G Koenning G A descriptive study of foot problems in children with juvenile rheumatoid arthritis (JRA) Arthritis Care Res 1994 7 144 150 10.1002/art.1790070308 7727554

[B47] Ferrari J A review of the foot deformities seen in juvenile chronic arthritis Foot 1998 8 193 196 10.1016/S0958-2592(98)90028-1

[B48] McCarney R Warner J Iliffe S van Haselen R Griffin M Fisher P The Hawthorne effect: a randomised, controlled trial BMC Med Res Methodol 2007 7 1 30 10.1186/1471-2288-7-30 17608932 PMC1936999

[B49] Lancsar E Louviere J Deleting ‘irrational’ responses from discrete choice experiments: a case of investigating or imposing preferences? Health Econ 2006 15 797 811 10.1002/hec.1104 16615039

[B50] Cairns J van der Pol M Lloyd A Decision making heuristics and the elicitation of preferences: being fast and frugal about the future Health Econ 2002 11 7 655 658 10.1002/hec.720 12369067

[B51] Bryan S Dolan P Discrete choice experiments in health economics: for better or for worse? Eur J Health Econ 2004 5 199 202 10.1007/s10198-004-0241-6 15452734

[B52] Kjaer T Bech M Gyrd-Hansen D Hart-Hansen K Ordering effect and price sensitivity in discrete choice experiments: need we worry? Health Econ 2006 15 1217 1228 10.1002/hec.1117 16786550

[B53] Ryan M Gerard K Using discrete choice experiments to value health care programmes: current practice and future research reflections Appl Health Econ Health Policy 2003 2 1 55 64 14619274

